# Adverse events and safety concerns among university students who misused stimulants to increase academic performance

**DOI:** 10.31744/einstein_journal/2024AO0895

**Published:** 2024-11-12

**Authors:** Joyce Emanuelle Moreira, Mariana Camile Las-Casas Rodrigues, Carlos Vinícius Teixeira Palhares, Thiago Henrique Caldeira de Oliveira, Gleisy Kelly Neves Gonçalves

**Affiliations:** 1 Faculdades Ciências Médicas de Minas Gerais Belo Horizonte MG Brazil Faculdades Ciências Médicas de Minas Gerais, Belo Horizonte, MG, Brazil.

**Keywords:** Central nervous system stimulants, Prescription drugs, Illicit drugs, Drug interactions, Students, Performance-enhancing substances, Learning, Health sciences, Universities, Academic performance

## Abstract

**Objective:**

To evaluate psychostimulant drug use among academics in the health area of a higher education institution in Minas Gerais, Brazil.

**Methods:**

We conducted an online cross-sectional study of 389 university students from various health-related fields. This study used a questionnaire to investigate the social and behavioral aspects associated with using psychostimulants.

**Results:**

The prevalence of psychostimulant use was 21%, primarily in men (23.07%), medicine students (19.70%), and psychology students (18.91%). Methylphenidate was the most commonly used drug (57%). Concomitant use of psychostimulants was reported in 37% and 35% of participants who started using them during graduation. A justifiable medical diagnosis was reported by 65% of the participants; however, a large portion had no indication for their use, and 77% acquired the medication without a medical prescription. The most frequently reported effects by users included lack of appetite (68%), tachycardia (58%), insomnia (43.5%), and agitation (50%). More than 70% of users also reported concurrent use of alcohol and illicit drugs, as well as depression and anxiety, which are contraindications for psychostimulant use. Additionally, 75% of students reported using psychostimulants for neuroenhancement purposes, with the majority (52%) perceiving their course performance as good and believing that it would be different without the drug (75.8%).

**Conclusion:**

Psychostimulant use in the study population revealed significant risks, including a lack of a valid diagnosis, unsupervised use, drug interactions, and side effects. Therefore, the data obtained in this study may contribute to the development of educational policies focused on preventing and controlling the indiscriminate use of these medications.

## In Brief

Moreira et al. demonstrated the concerning nonprescribed use of sychostimulants by health students, mainly among men, and by medical and psychology students. Significant risks include over-the-counter acquisition, concomitant use of alcohol/drugs, and adverse effects such as insomnia and tachycardia. Although 65% had a justifiable diagnosis, most were used to improve academic performance.

## Highlights

Psychostimulant use in the studied population revealed the following:

■ Lack of a valid diagnosis, unsupervised use, drug interactions, and adverse effects.
■ Concomitant use of alcohol and drugs and contraindications such as depression/anxiety.
■ Acquisition without prescription and use to improve academic performance.

## Graphical abstract

**Figure:**
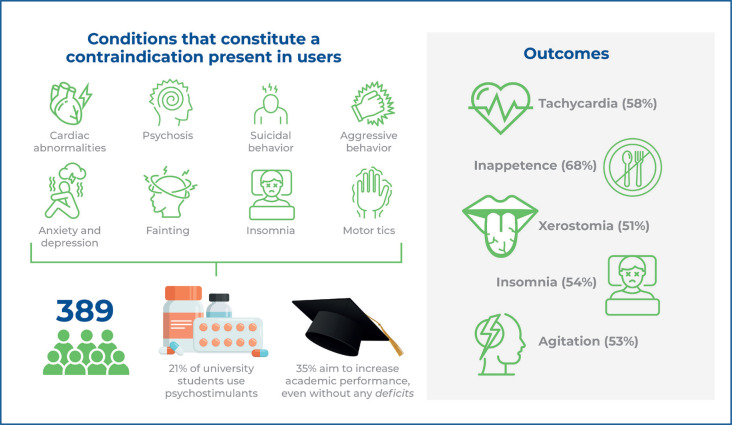

## INTRODUCTION

Psychostimulants are substances aimed to increase alertness, motivation, and concentration and are mainly used for the treatment of Attention Deficit Hyperactivity Disorder (ADHD). Psychostimulant drugs vary in their mechanisms of action and are generally associated with modulating dopamine (DA) through their pharmacokinetic and pharmacodynamic properties. Methylphenidate, the most widely consumed psychostimulant globally (according to the International Narcotics Control Board (INCB) 2018), acts on the Central Nervous System (CNS) by inhibiting the reuptake of dopamine (DA) and noradrenaline (NA) in the synaptic cleft. It also enhances the concentration and activity of alpha- and beta-adrenergic receptors associated with reward, excitement, motivation, and attention.^(1,2)^

However, individuals without any deficits use these substances not only for their intended purposes but also as cognitive enhancers. They seek improved performance in studies or work, even though scientific evidence does not support the advantages of psychostimulants in memory or learning. Healthy individuals typically obtain drugs for this purpose through a prescription, online without a prescription, or through family and friends.^(2,3)^

The misuse of these substances, which is primarily observed in academic circles, aims to enhance concentration and learning capacity. Factors such as high workload, academic pressure, limited leisure time, competitive environment, responsibility for patient care, pharmacological knowledge, and easier access to certain medications contribute to a higher prevalence of psychostimulant use among health students. Consequently, this population is constantly included as a target population in several studies that seek to analyze the variables of the indiscriminate use of psychostimulant drugs.^(3–7)^

In Brazil, studies have examined the association between students and indiscriminate psychostimulant drug use. The findings revealed that more than half of the users initiated their use during their academic lives, particularly during undergraduate studies. Notably, courses in the health field exhibited significantly higher prevalence rates than those in the humanities and exact sciences. Furthermore, students who inappropriately used psychostimulants stated that the medication's effects met the expectations of improved academic performance.^(5,6)^

The indiscriminate use of psychoactive substances leads to side effects, including sleep disorders, palpitations, anxiety, headaches, tremors, and gastrointestinal problems, which adversely impact cognition, physical well-being, and social functioning. Moreover, long-term abuse of psychostimulant drugs has been linked to chemical dependence. In this context, these medications have harmful repercussions that compromise the students’ quality of life.^(6,8)^

## OBJECTIVE

To evaluate psychostimulant drug use among students at a higher educational institution in Minas Gerais, Brazil.

## METHODS

This research, which consists of an observational cross-sectional study, was approved by the Research Ethics Committee of the *Faculdade de Ciências Médicas de Minas Gerais* (CAAE: 58896022.1.0000.5134; #5.664.926). The research participants were students in Medicine, Physiotherapy, Nursing and Psychology courses at an Educational Institution in Minas Gerais.

The study used an online form consisting of 30 questions, encompassing objective and discursive questions. It was made available to all students at the institution, regardless of their entry period. The questionnaire covered information such as participants’ age, course, and period, prevalence of psychostimulant use, non-use, commonly used medications, and possible concomitant use. Furthermore, the survey included questions regarding the frequency, side effects, initiation period, risk factors, contraindications, acquisition methods, knowledge level, medical monitoring, dependence, satisfaction level, association with anxiety and depression, and the impact of the COVID-19 pandemic and remote teaching. Students from the first period who consented to participate in the study and signed the Free and Informed Consent Form (TCLE) were included, whereas those under 18 years of age were excluded.

The sample size for data collection was calculated using a finite sample calculation with a 95% confidence interval and a sampling error of 5%, reaching the minimum necessary number of 385 participants.

The numerical data and discursive responses obtained were generated using the electronic form itself, stored in tables, and identified using codes to ensure the confidentiality and secrecy of the research. Quantitative data were analyzed by two researchers using computer tools (Microsoft Excel spreadsheets and statistical packages such as SPSS-15.0 for Windows) and presented in the form of tables, figures, and text.

To characterize the sample, we used simple frequencies and percentages for qualitative variables and medians with interquartile ranges for quantitative variables. Fisher's exact test and the χ^2^ test of independence were employed for data analysis, with a 5% significance level considered statistically significant.

## RESULTS

The study population comprised 389 university students who were divided into four health courses at an educational institution in Minas Gerais. Most individuals had studied medicine (71%), followed by physiotherapy (13%), psychology (9.5%), and nursing (6.7%). The sample was mainly composed of women (76%) with an average age of 21 years [interquartile range (IQR) 20.00-23.00] and included students in the first four years of their course. The main variables of interest are characterized below. For qualitative variables, simple frequency and percentage frequency are provided, and for quantitative variables, the median followed by interquartile range is given.

Table 1 presents a more targeted analysis of students who had already used or were currently using psychostimulant drugs (21%). Methylphenidate Hydrochloride (57%) and Lisdexamfetamine Dimesylate (47%) were the most frequently used medications, with 47% of the participants declaring that they used them daily and 46% sporadically. When asked about behavioral changes in the last 12 months, it was observed that the use of alcohol or drugs (77%), mental complaints (71%), and insomnia (51%) presented results with greater significance for this group. Most university students (57%) began medication use before college, whereas a notable proportion (35%) initiated use during their undergraduate studies. Among students who started medication use during college, 31% reported starting it within the first four years of their course.

**Table 1:** Profile and behavioral changes of users of psychostimulant drugs

Characteristics		n (%)
Has used or uses psychostimulant drugs		
	No	305 (79)
	Yes	83 (21)
Frequency of use of Methylphenidate Hydrochloride (Ritalin®)		
	Yes	47 (57)
	No	36 (43)
Frequency of use of Lisdexamfetamine Dimesylate (Venvanse®)		
	No	44 (53)
	Yes	39 (47)
Frequency of use of methylphenidate hydrochloride (Concerta®)		
	No	79 (95)
	Yes	4 (5)
Frequency of use of dextroamphetamine and amphetamine (Adderall®)		
	No	82 (99)
	Yes	1 (1)
Behavioral changes presented in the last 12 months Use of alcohol or drugs		
	Yes	64 (77)
	No	19 (23)
Heart abnormalities		
	No	72 (87)
	Yes	11 (13)
Fainting (epilepsy; seizures)		
	No	79 (95)
	Yes	4 (5)
Aggressive behavior		
	No	65 (78)
	Yes	18 (22)
Suicidal behavior		
	No	77 (93)
	Yes	6 (7)
Psychosis		
	No	81 (98)
	Yes	2 (2)
Motor tics		
	No	62 (75)
	Yes	21 (25)
Mental complaints (anxiety; depression; bipolarity; thought disorder)		
	Yes	59 (71)
	No	24 (29)
Insomnia		
	Yes	42 (51)
	No	41 (49)
Frequency of use of psychostimulant drugs		
	Every day	39 (47)
	Sporadically	38 (46)
	Once a week	4 (5)
	Uninformed	2 (2)
Period in which the use of psychostimulant drugs started		
	Before college	47 (57)
	During graduation	29 (35)
	In the childhood	5 (6)
	Uninformed	2 (2)

**Figure 1:**
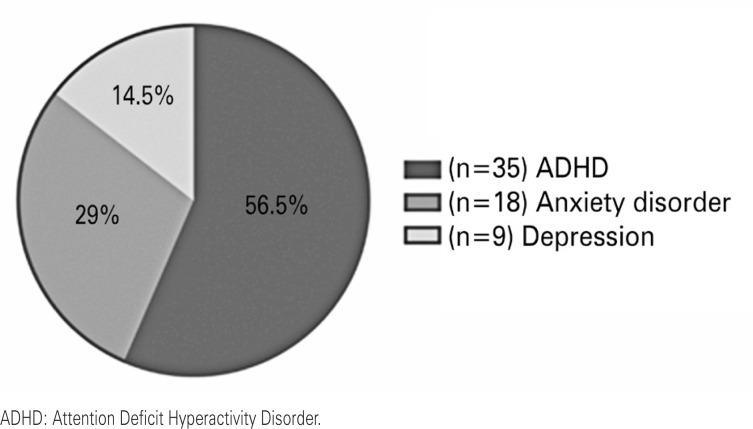
Diagnoses that justify the use of psychostimulant drugs

Regarding the users’ clinical profile (47%), the majority had a valid diagnosis of psychostimulant use. The most prevalent diagnoses were ADHD (56.45%), anxiety (29.03%), and depression (14.52%). Of the 62 diagnoses, individuals may have had multiple diagnoses simultaneously (Figure 1).

Furthermore, most students report using prescribed medications (77%) and having psychiatric monitoring (61%). However, 6% reported using higher dosages than recommended by professionals, and 61% admitted to searching the internet to determine the desired dosage. In this perspective, they were asked about the use of these medications to improve performance in studies, and 75% of students who used psychostimulants responded that they used them for this purpose. Of these, 22% declare that they are under the influence of drugs in less than 10% of academic activities, approximately 23% of students engage in around 30% of academic activities, and 20% participate in 100% of academic activities. Additionally, 11% participated in 50% of academic activities, while 9.8% engage in approximately 70% of academic activities (Table 2).

**Table 2:** Clinical profile of users of psychostimulant drugs

Characteristics		n (%)
Concomitant use of psychostimulant drugs		
	No	52 (63)
	Yes	31 (37)
Use of psychostimulant drugs with medical prescription		
	Yes	64 (77)
	No	18 (22)
	Not informed	1 (1)
Use psychostimulant drugs with dosage according to medical prescription		
	Yes	60 (72.3)
	Not applicable	15 (18.1)
Use in a dosage greater than recommended by the professional		5 (6)
Use in a dosage lower than recommended by the professional		1 (1.2)
Not informed		2 (2.4)
Follow-up with a psychiatrist		
	Yes	51 (61)
	No	32 (39)
Diagnosis that justifies the use of psychostimulant drug(s)		
	Yes	54 (65)
	No	29 (35)

Table 3 breaks down data related to the academic context of students who use drugs to improve performance in college (n=62). In this analysis, it was observed that more than half of the students classified their performance in the course as good and believed that if they did not use psychostimulant drugs, this response would be different, with the week preceding the assessment activities and tests being declared as the main period of use.

**Table 3:** Factors associated with the use of psychostimulant drugs in the academic context

Characteristics		n (%)
Use psychostimulant drugs at some point to enhance performance in studies, n=83 (100)		
	Yes	62 (75)
	No	21 (25)
Perception of performance in the undergraduate course, n=62 (100)		
	Good	32 (52)
	Average	20 (32)
	Very good	7 (11.2)
	Very bad	1 (1.6)
	Bad	1 (1.6)
	Not informed	1 (1.6)
They believe that their performance on the course would be different if they did not use psychostimulant drugs, n=62 (100)		
	Yes	47 (75.8)
	No	14 (22.6)
	Not informed	1 (1.6)
Specific situation that uses psychostimulant drugs, n=62 (100)		
	Week preceding assessment activities and tests	21 (33.9)
	Day of assessment activities and tests	9 (14.5)
	Only on the day of assessment activities and tests that you experience difficulty	7 (11.3)
	Not informed	25 (40.3)
Received information that encouraged to use psychostimulant drugs, n=62 (100)		
	No	42 (68)
	Yes, from a college friend	11 (18)
	Yes, from someone in the family	5 (8.1)
	Others	2 (3.2)
	Yes, through social media	2 (3.2)
Place of purchase of the drug, n=62 (100)		
	Pharmacy	24 (39)
	Doctor's office	21 (33.7)
	With a colleague	9 (14.5)
	With family	7 (11.2)
	Not informed	1 (1.6)
Performed an internet search to find out the dose necessary for the desired effect, n=62 (100)		
	Yes	38 (61)
	No	24 (39)

Most participants reported not being influenced to use psychostimulant drugs; however, 18% were encouraged by a college friend. The main place to purchase drugs was a pharmacy (39%), followed by a doctor's office (33.7%), and thirdly from a colleague (14.5%) (Table 3).

**Figure 2:**
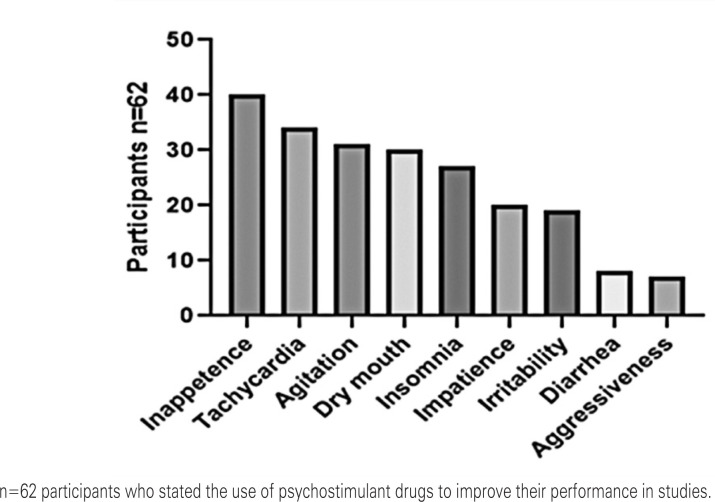
Side effects associated with the use of psychostimulant drugs to improve study performance

University students using psychostimulant drugs to enhance their academic performance were surveyed regarding their perception of side effects. The most reported effects by participants were increased alertness, heightened body activity, decreased appetite, tachycardia, agitation, dry mouth, insomnia, impatience, irritability, diarrhea, and aggressiveness (Figure 2).

Among participants using psychostimulant drugs to enhance academic performance, 68% (42 individuals) had a family history of mental disorders and comorbidities. Notably, depression (22), ADHD (14), anxiety (13), and insomnia (5) were the most frequently reported conditions. Additionally, cardiovascular (30), metabolic (25), and neurological (8) comorbidities were prevalent among the families of university students in this group. The study investigated university students’ perceptions of drugs, as the use of psychostimulant drugs for cognitive neuroenhancement is common in academia. However, contrary to beliefs, no evidence supports the notion that these medications enhance attention, memory, and learning capabilities. Additionally, no relationship was found between the COVID-19 pandemic and the initiation of medication use. Regarding satisfaction with the academic performance-enhancing effects, most students (84%) reported being satisfied. Sixty-nine percent of students who have used or are currently using psychostimulant drugs for academic performance improvement are aware of peers at the same institution who have the same habit, underscoring the study's significance and the relevance of this topic.

## DISCUSSION

The off-label use of medicines refers to their use in situations not registered in the leaflet, such as the use of unusual dosages for unapproved therapeutic purposes and indications different from those recommended.^(9)^ Furthermore, users often overlook the side effects and potential for chemical dependency associated with the abuse of these substances. This leads to unrealistic expectations and contributes to the significant public health problem it poses in Brazil and worldwide.^(5)^

In this study, most of the analyzed students are female, studying medicine and physiotherapy, with an average age of 20 to 23 years. However, the study observed higher prevalence of psychostimulant medication use among males (23.07%), which aligns with Meiners et al. findings^(10)^ (18.3%) and different from the study by Cândido et al.,^(11)^ where it was found a higher prevalence of psychostimulant use in females (67%). Thirty-five percent (35%) of students started using such drugs during their undergraduate studies, with a higher prevalence among medical (19.70%) and psychology (18.91%) students, making a total of 77.1% and 8.9% of students who have already used psychostimulants, respectively. The result found was similar to Webb's study et al.,^(12)^ conducted with medical students from the United States (15%), and Silva et al.,^(5)^ also performed at a Higher Education Institution in Minas Gerais, where it was found a higher prevalence of use of psychostimulants among medical students (37.3%, totaling 66.7%) compared to administration and accounting courses, law, nursing, pharmacy, and physiotherapy.

The heavy workload, demanding content, performance expectations, and responsibility in health courses, including practical classes and patient care, place a significant burden on students. As a result, this audience often turns to pharmacological neuroenhancement medications to enhance cognitive performance, motivation, attention, and alertness.^(13–16)^ Another important factor to be considered is the relationship with self-medication, which may be predisposed due to greater knowledge about medications. In this way, these university students form a risk group for the inappropriate use of stimulant substances, which also demonstrate an obvious risk to individual health, considering the impact these drugs have on the human body.^(3,17)^

The prevalence of the use of psychostimulants found was 21%, higher than that found by Meiners et al.^(10)^ in the Federal District (15%), by Cândido et al.^(11)^ at *Universidade Federal de Minas Gerais* (9.8%), and by Bucher et al.^(18)^ in Philadelphia (14%). Among the 21% who reported using psychostimulants, 57% specifically mentioned using Methylphenidate, which is the most commonly used medication among academics. Within the Methylphenidate user group, 65% had a medical diagnosis justifying its use, primarily for ADHD, while 35% did not. Therefore, concluding that the use in this group was solely for cognitive enhancement is uncertain. Methylphenidate marketing is regulated by the National Health Surveillance Agency (ANVISA - *Agência Nacional de Vigilância Sanitária*),^(19)^ requiring a special prescription. However, the study revealed that 22% of users obtain it without a medical prescription, primarily from colleagues. This highlights a failure in public policies aimed at controlling its usage, as well as the existence of an underground market. This study did not collect data on the average dose or types of drug release. In a similar way, Candide's studies et al*.*^(11)^ also reported that students who purchased psychostimulants without a medical prescription had their indiscriminate use recommended by friends, with the pharmacy being the main place of purchase. Furthermore, among the 77% who use the medicine with a medical prescription, 7.2% do so incorrectly.

Concomitant use of psychostimulants was observed in 37% of the academics participating in the study, with one participant reporting the use of Dextroamphetamine and Amphetamine, a central nervous system stimulant belonging to the phenylethylamine class and illegal in Brazil. Incorrect use of these medications can lead to dysregulation of the attentional system, predisposing individuals to attention deficits. Additionally, activation of reward areas may drive a constant search for pleasure, leading to an increased dose requirement, addiction, and eventual use of illicit drugs.^(2)^

Regarding the side effects perceived after the administration of medications, the results demonstrate that most of the effects cited in the literature occurred with participants who use or have already used psychostimulants, with an emphasis on inappetence (68%), tachycardia (58%), agitation (53%), xerostomia (51%), and insomnia (54%). Higher numbers were found compared to the study of Nasário et al.,^(20)^ who reported a frequency of 4.1% of insomnia, 6.2% of agitation, and 7.8% of tachycardia, and to those found by Meiners et al.,^(10)^ in which 40% of participants reported tachycardia and 36.7% xerostomia.

In addition to the observed side effects, 77% of study participants reported concurrent alcohol and drug use in the past 12 months. This association can increase medication toxicity, intensify its effects, and potentially lead to serious side effects. Therefore, such a combination is contraindicated. Without psychiatric analysis, classifying alcohol and drug use as substance use disorders is difficult. In this case, it only indicates the frequency of alcohol and drug use. A similar result was found in the study of Cândido et al.,^(11)^ in which 55% of participants reported using alcohol and 28.6% used illicit drugs. Psychostimulants, especially methylphenidate, are also contraindicated for patients suffering from anxiety or agitation, severe depression, suicidal ideas, tics, psychoses, hyperthyroidism, and cardiovascular diseases.^(5)^ However, academics who use psychostimulants reported behavioral changes that constitute contraindications, such as mental complaints, anxiety, and depression (71%), insomnia (51%), motor tics (25%), aggressive behavior (22%), cardiac abnormalities (13%), suicidal behavior (7%), fainting (5%), and psychosis (2%).

Medical students commonly experience negative effects such as anxiety, bad mood, anguish, sadness, and fear more frequently than positive effects. These feelings can predispose them to symptoms and mental disorders, underscoring the need for educational measures on drugs. Health academics, in most studies, have the highest prevalence of psychostimulant abuse.^(21)^

The students surveyed showed a 75% prevalence of indiscriminate use of drugs to enhance performance. Of these, 42% do not have a medical diagnosis that justifies the use of psychostimulants, and 33.9% only use them in the week preceding assessment activities and tests. Furthermore, more than half classify their performance on the course as good and believe that it would be different if they did not use the drugs; however, academic performance parameters were not used in the study. Published studies discuss the real effects of psychostimulants and open discussions regarding improving academic performance. A work performed by Nasário et al.^(20)^ presents data regarding the average performance of academics with and without the use of methylphenidate, which corroborates the hypothesis that the effect of using methylphenidate by people who do not have a deficit related to dopamine and/or noradrenaline levels seems to be related to feelings of well-being and a self-perception of cognitive improvement. This research also assessed the reported frequency of psychostimulant drug misuse and found similar results, suggesting a practice that may be associated with cognitive enhancement and can vary based on academic demands. In this group, a 68% prevalence of a family history of mental disorders, such as depression, ADHD, anxiety, and insomnia, is observed. Comorbidities in the family history include cardiovascular diseases (Systemic Arterial Hypertension, Heart Failure), metabolic diseases (Diabetes Mellitus, Obesity) and neurological, which are also related to possible contraindications to the use of psychostimulants. These diseases are common and can often be asymptomatic, resulting in delayed diagnosis. They have multiple causes, including modifiable risk factors like lifestyle habits and non-modifiable factors like genetics. Hence, emphasizing the study's finding of off-label and indiscriminate use of psychostimulants among academics who have a family risk of chronic diseases is crucial due to its potential safety risks.

Several limitations must be considered when interpreting the results of this study. The cross-sectional design means that inferring causal relationships between prescription stimulant use and other factors is not possible. Self-report measures may introduce memory and social desirability biases, while the recruitment method may introduce selection bias, as students who used prescription stimulants or illicit drugs were less likely to participate. The study's limitations includes a non-representative sample, as it only evaluated students from a single institution using a convenience sample. The data collection relied on voluntary participation through in-person questionnaires at the institution and on social media. This undermines the sample uniformity across the evaluated courses. The study relied on participants to differentiate between using prescription stimulants for studying and for recreational or other purposes, with the survey explicitly reminding participants of this distinction. Therefore, the findings should be interpreted cautiously, considering the possibility of reduced prevalence of use due to non-inclusion in the research.

## CONCLUSION

A significant prevalence of psychostimulant use was observed among health field students, with the majority lacking medical indications for its use. The results show that participants experienced most of the side effects mentioned in the literature, and over half of the students reported behavioral changes that are contraindications for drug use. This highlights the detrimental effects of indiscriminate drug use on individual health. Furthermore, more than half of participants reported using the medication to enhance academic performance, and the majority perceived it as effective. This presents significant challenges in combating this practice effectively. Health courses should prioritize discussions on the use of psychostimulants, considering that students in these courses are at risk and may encounter similar situations in their future professional environments, including being prescribers of such medications. The growing use of psychostimulant drugs for academic performance necessitates addressing the risks of adverse effects. This highlights the importance of conducting further studies and reflecting on the issues raised to develop effective intervention strategies. The profile of psychostimulant drug use in the study population revealed significant risks, including: unjustified medical diagnosis; unsupervised use; concurrent use of substances resulting in potential drug interactions; and side effects like tachycardia, insomnia, and agitation.
